# Hyperthermic intrathoracic chemotherapy for the treatment of malignant pleural effusion caused by breast and ovarian cancer: A systematic literature review and pooled analysis

**DOI:** 10.1111/1759-7714.14361

**Published:** 2022-02-22

**Authors:** Ioannis Karampinis, Anna Dionysopoulou, Christian Galata, Katrin Almstedt, Maurizio Grilli, Annette Hasenburg, Eric D. Roessner

**Affiliations:** ^1^ Division of Thoracic Surgery, Academic Thoracic Center University Medical Center Mainz, Johannes Gutenberg University Mainz Germany; ^2^ Department of Obstetrics and Gynecology University Medical Center Mainz, Johannes Gutenberg University Mainz Germany; ^3^ Department of Library and Information Sciences, University Medical Center Mannheim, Medical Faculty Mannheim Heidelberg University Mannheim Germany

**Keywords:** breast cancer, HITOC, intrathoracic chemotherapy, malignant pleural effusion, ovarian cancer

## Abstract

**Objectives:**

Breast and ovarian cancer account for over 30% of malignant pleural effusions (MPEs). Treatment of the metastatic disease requires control of the MPE. Even though primarily symptomatic, the treatment of the MPE can potentially affect the oncological course of the disease. The aim of this review is to analyze the effectiveness of intrathoracic chemotherapy in the treatment of MPE caused by breast and ovarian cancer.

**Methods:**

A systematic literature research was conducted up until May 2021. Studies published in English on patients undergoing either surgical or interventional intrapleural chemotherapy were included.

**Results:**

Thirteen studies with a total of 497 patients were included. Analysis was performed on 169 patients with MPE due to breast cancer and eight patients with MPE secondary to ovarian cancer. The pooled success rates of intrathoracic chemotherapy for controlling the MPE were 59.1% and 87.5%, respectively. A survival analysis was not possible with the available data. The overall toxicity of the treatment was low.

**Conclusions:**

Intrathoracic chemotherapy achieves symptomatic control of the MPE in 59.1% of patients with metastatic breast cancer and 87.5% of patients with metastatic ovarian cancer. This is inferior to other forms of surgical pleurodesis. Data from small case series and studies on intraperitoneal chemotherapy show promising results. However, formal oncological studies on the use of intrathoracic chemotherapy for metastatic breast or ovarian cancer are lacking. Further prospective pilot studies are needed to assess the therapeutic oncological effects of this treatment.

## Graphical abstract

A systematic literature research was conducted up until May 2021 to analyze the effectiveness of intrathoracic chemotherapy in the treatment of malignant pleural effusion (MPE) caused by breast and ovarian cancer. Analysis was performed on 169 patients from 13 different studies with MPE due to breast cancer and eight patients with MPE secondary to ovarian cancer. The pooled success rates of intrathoracic chemotherapy for controlling the MPE were 59.1% and 87.5%, respectively. The overall toxicity of the treatment was low.

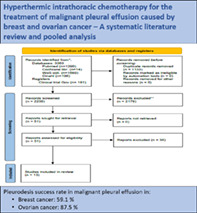

## INTRODUCTION

Every third patient with malignant pleural effusion (MPE) suffers from either metastatic breast or ovarian cancer.^1^ The need for treatment for MPE is usually triggered by the patient's symptoms. Simple aspiration, interventional drainage or surgery have all been proposed as treatment options, depending on the symptoms and the recurrence rate of the effusion.^2^ There is no clear correlation between the severity of the symptoms and the size of the effusion.^3^ However, symptomatic pleural effusions are more likely to be bigger and tend to recur more often.

The diagnosis of MPE has been associated with poor prognosis, which ranges from a few weeks to several months.^4^ It is therefore common practice that patients with confirmed MPE only qualify for palliative treatment. However, the current trend in oncology is towards aggressive treatment even in metastatic disease.^5^, ^6^ Intrathoracic chemotherapy has been proposed as a treatment option for metastatic pleural disease and has been used to treat several malignancies, including mesothelioma, lung cancer, thymic malignancies, and peritoneal surface malignancies.^7^, ^8^, ^9^

A recently published study described the influence of MPE in the survival rate of patients with advanced ovarian cancer and established an algorithm for approaching these cases.^10^ According to the proposed algorithm, a thoracoscopy needs to be performed initially to determine the presence or absence of macroscopic pleural disease. Depending on the findings, the authors either proceed with debulking or end the procedure and the patient receives induction chemotherapy followed by thoracic debulking. As a last step the patients undergo abdominal cytoreduction. By following this treatment algorithm, the authors managed to increase the number of patients that qualify for aggressive treatment whilst avoiding overtreating those with advanced disease.

The purpose of our study was to analyze the role of intrathoracic chemotherapy in the treatment of MPE caused by breast and ovarian cancer.

## MATERIALS AND METHODS

The objective of this review is to determine the rate of local control of MPE in patients with metastatic breast or ovarian cancer that undergo intrathoracic chemotherapy. Furthermore, we aim to determine the impact of this treatment on the survival rate of these patients.

All studies reporting results on local control of the MPE or reporting survival data were included. Given that the actual recurrence rate and therefore local control rate of the effusion for metastatic breast and ovarian cancer are unknown, studies were included regardless of the number of patients.

### Systematic literature research

A computer‐based literature search was performed (MG) up until May 14, 2021 in several databases, including the Cochrane Central Register of Controlled Trials (CENTRAL), the Cochrane Database of Systematic Reviews (CDSR) from The Cochrane Library, MEDLINE (1966 to present), Cinahl (1981 to present), and Web of Science (1945 to present). Reference lists of retrieved articles were scanned for further eligible trials (backward search) and citations of identified trials were checked for inclusion (forward search). Search strategies included proper combinations of the MeSH terms “HITHOC”, “breast cancer”, and “ovarian cancer.” The search was not limited by publication type. Non‐English papers were excluded. The complete search strategy is available as Supporting Information.

Two reviewers (A.D. and I.K.) independently performed the extraction of data from the included studies. The findings of the two reviewers were controlled for concordance and disagreements were resolved with discussion and detailed analysis of the trials and the data.

The success rate of intrathoracic chemotherapy for controlling the MPE was estimated as the proportion of patients with either complete or partial response to the total number of patients that was treated. A quality/bias assessment was not performed due to the low or very low quality of the included studies.

## RESULTS

**FIGURE 1:**
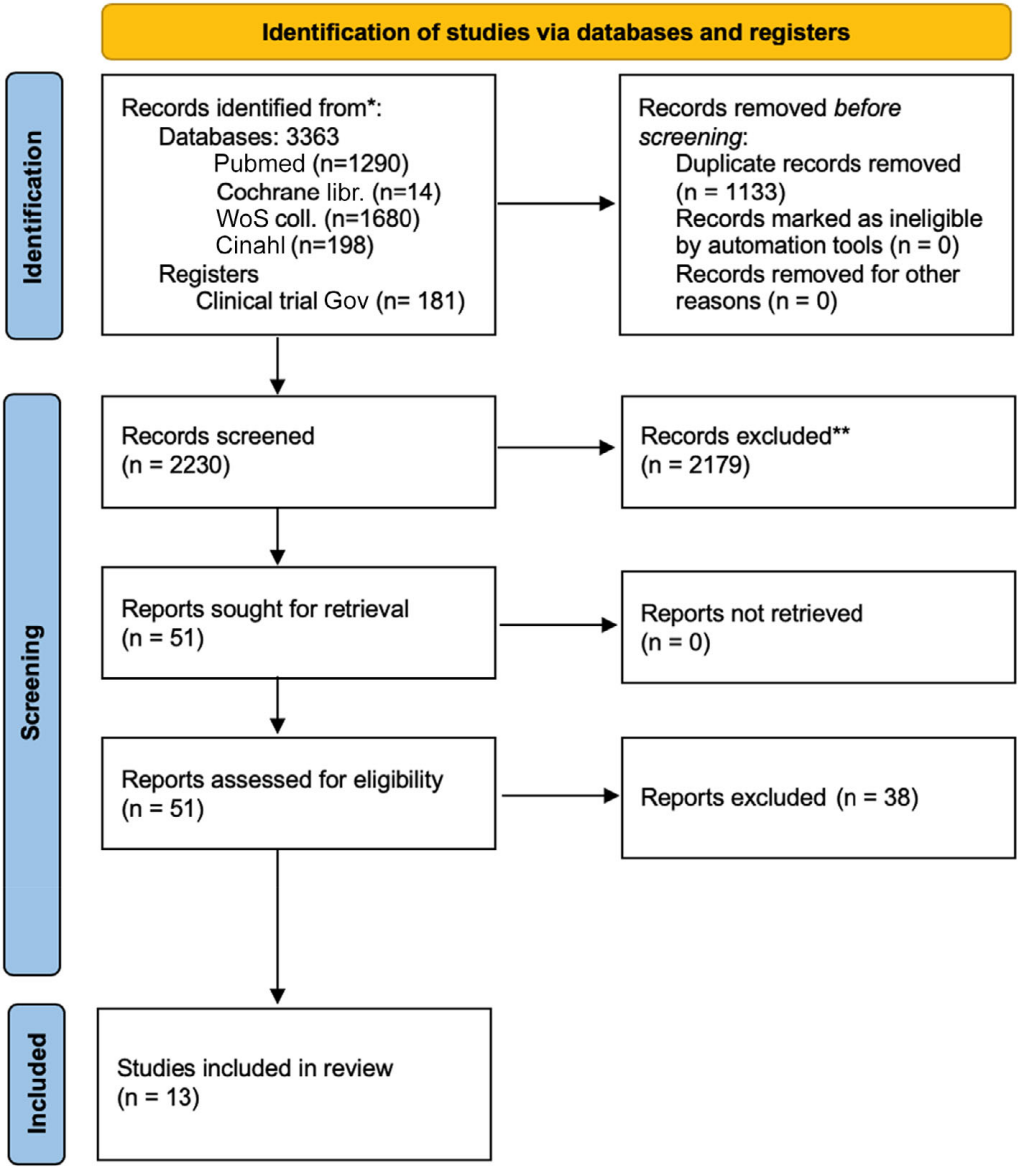
PRISMA flowchart

The database search provided 3363 references. After deduplication, 2230 studies were available for screening (Figure 1). A total of 2179 records were excluded and 51 abstracts were assessed for eligibility. In addition, 38 were excluded and 13 studies were included in the review. Hand searching of the references of the included studies provided one extra reference, which was eventually excluded.

Studies on intrathoracic chemotherapy for malignancies other than breast or ovarian cancer, studies on patients undergoing other forms of pleurodesis, as well as studies analyzing the impact of nonchemotherapy associated intrapleural treatment were excluded.

The included studies are listed in Table 1. Six studies were retrospective, one of which was a multicentre retrospective study,^11^ four studies were prospective, two were case reports, and one was a randomized controlled trial. The 13 studies included a total of 497 patients, and 286 of these patients were treated for MPE caused by breast or ovarian cancer.

**TABLE 1:** Included references

Author	Year	Study type	Pat. Number	Disease	Procedure
Fracchia^12^	1970	Retrospective	138	BC	IC drain
Markman^13^	1984	Retrospective	4	1 BC/3 OC	IC drain
Contegiacom^14^	1987	Retrospective	21	BC	IC drain
Rusch^15^	1991	Prospective	46	8 BC/5 OC^a^	IC drain
Kan^11^	1993	Retrospective	67	BC	IC drain
Aasebo^16^	1997	Prospective	30	20 BC/1 OC^a^	IC drain
Shoji^17^	2002	Prospective	22	2 BC^a^	VATS + port
Mitamura^18^	2009	Case report	1	OC	IC drain
Jones^19^	2010	Prospective	15	4 BC/1 OC^a^	VATS + IC drain
Singh^20^	2014	Retrospective	4	OC	VATS + cytoreduction
Feng^21^	2017	Retrospective	80	6 BC^a^	VATS + washout
Jun^22^	2017	Case report	1	OC	VATS + cytoreduction
Zhang^23^	2021	RCT	84	19 BC	IC drain

Abbreviations: BC, breast cancer; IC, intercostal drain; OC, ovarian cancer; VATS, video‐assisted thoracoscopic surgery.

^a^ Means that not all patients provided in the fourth column had breast or ovarian cancer, e.g. for Rusch et al. 13 of 46 patients had either breast or ovarian cancer, etc.

In 12 studies, the endpoint was local control or recurrence of the MPE. The purpose of the 13th study was to examine the effectiveness of repeated intrapleural chemotherapy through a port system and report only the survival rate of the included patients.^17^ Four studies reported no toxicity after the intrapleural chemotherapy.^14^, ^17^, ^20^, ^22^ In the complete cohort of 497 patients, 11 cases of myelosuppression and another 18 cases of other grade 3/4 toxicity were described. One study established the levels of intrapleural toxicity of docetaxel.^19^ By administering doses between 50 and 125 mg/m^2^ the authors achieved a pleural exposure which was 1000 times higher than the systemic exposure, whilst observing a single dose‐limiting toxicity in a patient that had received 50 mg/m^2^. All patients that received doses of 100 mg/m^2^ or higher achieved complete resolution of the MPE.^19^

The cytotoxic agent, nitrogen mustard or thio‐TEPA, was administered intrapleural in one study.^12^ In seven studies a platin‐based chemotherapy was used and further two studies combined platin‐based chemotherapy with paclitaxel.^18^, ^20^ The other studies used different combinations of chemotherapy. One study that compared intrapleural cisplatin alone and intrapleural cisplatin combined with external application of mirabilite rhubarb in a randomized setting concluded that the second group had a significantly higher success rate for achieving local control of the disease. Six studies excluded patients who were receiving concurrent systemic chemotherapy while in two other studies all patients were receiving systemic chemotherapy.

**FIGURE 2:**
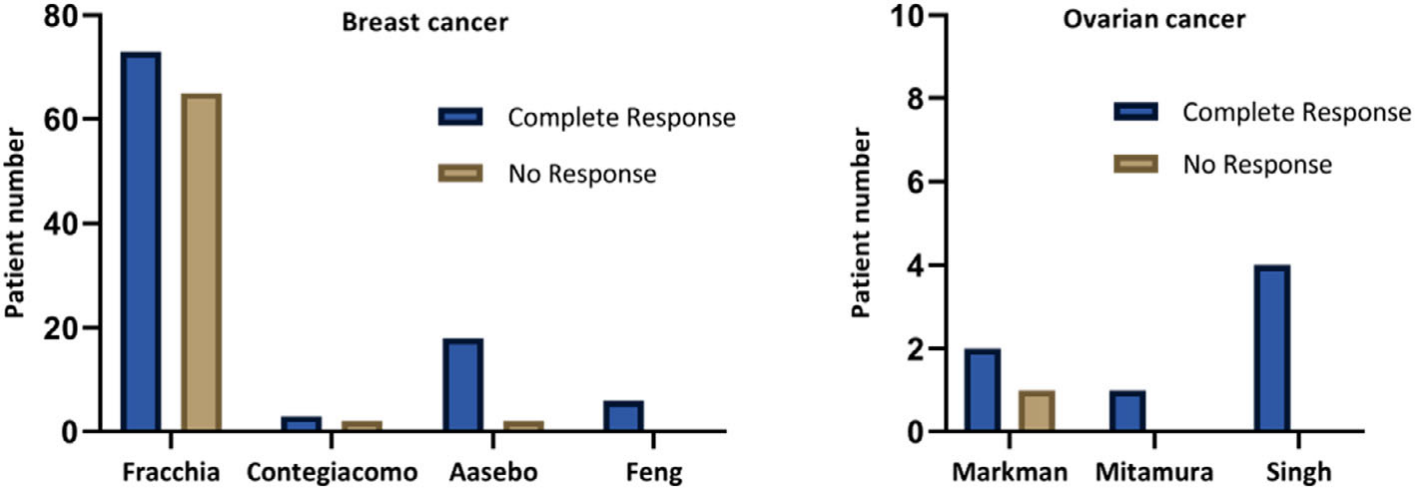
Success rate of individual studies in achieving local control of the MPE

The median time point that the efficacy of the intrapleural chemotherapy was assessed was 4 weeks (range 3–8 weeks) and was reported in seven studies. The pooled success rate of intrapleural chemotherapy for controlling the MPE was 59.1% for breast cancer patients and 87.5% for ovarian cancer. The success rate in the individual studies is presented on Figure 2.

## DISCUSSION

MPE is a debilitating diagnosis that is associated with severe symptoms, the need for regular interventions or surgical therapy, and delay in systemic treatment. The standard management of MPE is largely dependent on the symptoms and the prognosis of the patient. Fit patients with an estimated prognosis of a few months or longer usually receive surgical treatment, whereas patients with a poor performance status or with a poor prognosis are usually treated conservatively with pleural taps or indwelling pleural catheters.^2^

Intrathoracic chemotherapy for the treatment of MPE was first described in 1955.^24^ In advanced abdominal tumors, extensive tumor resection (cytoreduction) followed by intraoperative administration of local chemotherapy with simultaneous warming of the abdominal cavity is used to improve survival in selected patients. This concept of hyperthermic intraperitoneal chemotherapy (HIPEC) was initially developed to treat metastatic disease of the peritoneum and was later transferred to the chest cavity (hyperthermic intrathoracic chemotherapy, HITHOC), mainly to treat malignant mesothelioma.^25^ Since then, numerous studies on the use of intrathoracic chemotherapy have been published. However, most of the literature are case series, feasibility studies, and retrospective studies with small numbers of patients. When administered locally, the chemotherapy can be applied in much higher doses than systemically tolerable, leading to both a sclerosing and a cytotoxic effect.^19^ The main tumor types that intrathoracic chemotherapy has been implemented on are lung cancer, mesothelioma, and thymic malignancies. Despite the high prevalence of MPE in patients with metastatic breast and ovarian cancer, limited data exist on the use of intrathoracic chemotherapy as a treatment option for these patients.

In this study we reviewed the impact of intrathoracic chemotherapy for the treatment of MPE caused by breast or ovarian cancer. A total of 169 patients with breast cancer and eight patients with ovarian cancer were pooled to calculate a local control rate of 59.1% for breast cancer and 87.5% for ovarian cancer at 4 weeks after treatment. Survival data was not available for analysis.

The pooled success rate of 59.1% is inferior to other forms of surgical pleurodesis, such as talc pleurodesis in patients with metastatic breast cancer.^26^ It is therefore reasonable to suggest the use of talc in case only pleurodesis is attempted. For patients with metastatic ovarian cancer the success rate of intrathoracic chemotherapy for achieving pleurodesis was higher than average. However, only eight patients from three different studies were utilized to calculate this percentage, and therefore this may not be representative of the true value.

It is arguably not a worthwhile venture to investigate further trials or studies to research the use of intrathoracic chemotherapy in achieving pleurodesis in patients with breast and ovarian cancer. However, given the low level of toxicity, the incapacitating effects of MPE, and the current trend towards more aggressive treatment of several malignancies, it is certainly worth doing further clinical trials to test its oncological efficacy.

Questions that need to be clarified in upcoming studies should be the selection criteria for this treatment, the chemotherapeutic agents that should be applied as well as the type of surgery that is indicated. The combination of surgical cytoreduction with hyperthermic chemotherapy has been proven to be the most effective from the oncological point of view for other malignancies (thymic tumors, malignant pleural mesothelioma). On the other hand, newer, less invasive procedures like the pressurized intrathoracic chemotherapy are currently gaining approval in this field.

### Limitations

This review/pooled analysis has several limitations. First, there is a high level of heterogeneity in the included studies, especially with regards to the oncological status/treatment stadium of the included patients, the protocol, the agents used for intrathoracic chemotherapy, the follow‐up, and the outcome assessment algorithms. Second, this review covers a period of 50 years, which has a significant impact on the interpretation and the comparability of the results of the included studies.

Third, this analysis pools together studies on intrathoracic chemotherapy through an intrapleural catheter and studies on intrathoracic chemotherapy following thoracoscopic cytoreductive surgery. The purpose of the first studies is obviously palliation/pleurodesis while the purpose of the second ones could be potentially curative treatment, although only limited disease can be effectively resected through thoracoscopic cytoreduction. The effect of a pleurectomy/decortication followed by intrapleural chemotherapy is not presented in this review since no proper evidence exists so far. Nevertheless, the pooled results of this analysis can offer a better overview of the available data and direct future studies, rather than being used as absolute numbers, since they may deviate significantly from the truth.

## CONCLUSION

MPE is a common complication of metastatic breast and ovarian cancer. Treating the effusion is often required to ease the symptoms of the patients, but might as well affect the oncological outcome of the disease. Compared to other nonsurgical forms of pleurodesis, intrathoracic chemotherapy has a comparable efficacy in achieving pleurodesis. However, it is inferior to surgical pleurodesis and has a higher rate of adverse events. Results from reports and small case series demonstrate that even thoracoscopic cytoreduction followed by intrathoracic chemotherapy can have a positive impact on survival as part of a multimodality oncological treatment on selected patients and should therefore be evaluated in further prospective pilot studies.

## CONFLICT OF INTEREST

The authors have no competing interests to declare.

## AUTHOR CONTRIBUTIONS

I.K. was involved in the conception of the project, extraction and analysis of the data, and drafting and revision of the manuscript. A.D. was involved in the conception of the project, performed the extraction, acquisition, and analysis of the data, and drafted the manuscript. C.G. was involved in the processing of the data and the data analysis. K.A. was involved in the processing of the data and the data analysis. M.G. performed the systematic literature research. A.H. was involved in the conception of the project and critically revised the manuscript for important intellectual content. E.R. was involved in the conception of the project and critically revised the manuscript for important intellectual content. I.K., A.D., and C.G. had full access to the data presented in this study.

## Data Availability

The datasets generated during the current study are not publicly available since the main part is included in this article. The complete database is available from the corresponding author on reasonable request.
